# Diversity in psychiatry education and patient and public involvement: roundtable analysis

**DOI:** 10.1192/bjb.2024.106

**Published:** 2026-02

**Authors:** Miriam Stanyon, Nagina Khan, Analia Buckley, Naina Patel, Kirit Mistry, Karl Ryan, Subodh Dave

**Affiliations:** 1 Derby Psychiatry Teaching Unit, Derbyshire Healthcare NHS Foundation Trust, Derby, UK; 2 Centre for Health Services Studies, University of Kent, Canterbury, UK; 3 NIHR Research Support Service Hub, University of Leicester and Partners, Leicester, UK; 4 South Asian Health Action, Leicester, UK

**Keywords:** Education and training, patients, qualitative research, stigma and discrimination, transcultural psychiatry

## Abstract

Patient involvement in psychiatry education struggles to be representative of the patients that doctors will treat once qualified. The issues of mental health stigma, cultural perspectives of mental health and the unique role of teaching, required exploring to establish the barriers and facilitators to increasing the diversity of patients involved in psychiatry education. To explore the causes of this lack of representation, a roundtable event with 34 delegates composed of people with lived experience of mental health issues, people from underserved communities, academics, mental health professionals and charity representatives met to discuss the barriers to involvement in psychiatry education and possible solutions. Themes were further developed in a context expert focus group. Notes from the roundtable and focus group were analysed and developed into recommendations for medical schools and mental health professional teaching departments.

## PPI in healthcare education

Patient and public involvement (PPI) is foundational to healthcare education. In many nations, PPI is required by policy in the education of healthcare professionals,^1–3^ where they are valued as experts by experience and compliment the clinical expertise of clinicians.^4–6^ PPI in healthcare education is fast moving toward the gold-standard of co-production.^7,8^ However, there are still challenges with ensuring that those involved reflect the diversity of the general population. Like PPI in health research, those who get involved in healthcare education are often ‘White, middle-class, highly educated, retired professionals’ and are frequently involved in multiple PPI opportunities.^9–11^

## Barriers to PPI

Diversity in psychiatry PPI is particularly important as there are added barriers of mental health sigma and differences in cultural perceptions of mental illness.^12,13^ The increasing globalisation of healthcare means that a physician in any country will be treating patients from varied cultural backgrounds.^14,15^ There are also parallels with the international discourse on underserved communities^16^ and health inequalities.^17^ Recommendations helping to widen diversity in PPI in healthcare research^18^ are a good starting point for psychiatry education; however, we sought greater detail of the interacting barriers of mental health, culture and the vulnerability that the teaching role brings.

## PPI in Derby PTU

The teaching team at Derby Psychiatry Teaching Unit (PTU) in the East Midlands, UK, became aware that the patients involved in our teaching were not representative of the population in Derby. We ran a roundtable event, bringing together researchers, clinicians, charity representatives and members of the public from underserved communities with lived experience of mental health conditions, with the aim of developing recommendations for how to increase the diversity of PPI in psychiatry education.

## Method

### Recruitment

A roundtable event was held in the East Midlands, UK. Local charities and community groups, research departments and PPI groups were informed. One charity worker in the South Asian community was funded to gather delegates and facilitators for workshops. Delegates registered online or by contacting the project manager. Delegates could attend in person or online via Microsoft Teams.

### Data collected

When booking, delegates were asked for their role (public contributor, researcher, clinician or charity representative), whether they had lived experience of mental health conditions and whether they identified with an underserved community.

During the workshops facilitators took notes. The online workshop used the ‘chat’ function for delegates to record their thoughts. Verbal informed consent was obtained from all delegates for their comments to be used anonymously in publications and presentations.

### Procedure

On arrival, delegates were assigned to a group containing four to eight people, including a trained facilitator with lived experience of a mental health condition and an academic co-facilitator. All facilitators had previously attended a 90 min online training session covering the aims, structure of the day, ethos of partnership in PPI, method of note-taking and tips for facilitating.

**Fig. 1:**
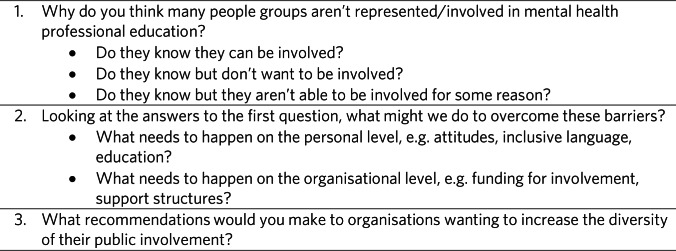
Questions for the roundtable.

Groups were at least 50% composed of public contributors. After each workshop, facilitators presented a summary of discussions. Each group took part in two workshop sessions during the day. This paper discusses the outcomes of the first workshop focusing on the barriers and facilitators to involvement in psychiatry education for those from underserved communities. The topic guide can be found in Fig. 1. The workshop lasted approximately 1 h.

At the end of the day, all notes and field notes were collected.

### Ethical considerations

Ethical approval was not deemed necessary as the roundtable and follow-up focus group were classed as engagement and involvement activity rather than research. All delegates had the opportunity to be reimbursed for travel. Public contributors were reimbursed for their time. Delegates who received benefits payments were given a letter to explain the reimbursement. Delegates were encouraged to take a break or speak to one of the clinicians on the event organising team if needed, and given contact information for support following the event.

### Follow-up focus group

An in-person focus group was conducted with six lived experience educators at Derby PTU who were experienced in the context of PPI in psychiatry education. The aim was to explore how the findings of the roundtable could be applied to their roles in psychiatry education, further developing recommendations for increasing the diversity of PPI. The topic guide from the workshop was a starting point for discussion but progressed inductively, with no predefined themes. Data was recorded by the facilitator via note-taking and participant observation. The focus group lasted approximately 1 h. Notes taken by the facilitator were incorporated into the data analysis from the roundtable.

### Analysis

The notes from each workshop and the follow-up focus group were analysed thematically, using an inductive thematic analysis process proposed by Braun and Clarke.^19^ Notes were read and re-read to familiarise the analysts with the data. Provisional codes were developed and applied to the rest of the data. These were then grouped into themes. These themes and codes were reviewed across the whole data-set, named and defined. The data within themes was summarised and data arranged into tables displaying the summaries and recommendations resulting from the data. Facilitators to engagement were rephrased as recommendations.

## Results

### Participants

Thirty-four people attended the roundtable event. The characteristics of delegates across the groups are represented in Table 1. Many delegates spanned more than one category of role, protected characteristics and experience of mental health. The intersectionality of many of the delegates was a key strength of the event.

**Table 1:** Delegates according to roles, protected characteristics and group

	Table 1	Table 2	Table 3	Table 4	Table 5	Table 6 (Virtual)	Totals
Roles							
Member of the public	2	4	3	5	2	1	17
Mental health clinician		1		1	1	4	7
Researcher	1				1	5	7
Charity representative	1		1		1		3
Ethnicities/protected characteristics							
African Caribbean		1			2		3
Asian	1	1	2	2	2	5	13
Eastern European				1			1
South American		1					1
LGBTQ^+^	1					1	2
Autism diagnosis		1					1
Long-term health condition				1		1	2
Dementia diagnosis			1	1			2
Total from underserved communities	2	4	3	3	4	7	23
Experience of mental health condition	2	4	2	2	2		12
Total delegates in group	4	5	4	6	5	10	34

### Barriers and facilitators to involvement in psychiatry education

**Fig. 2:**
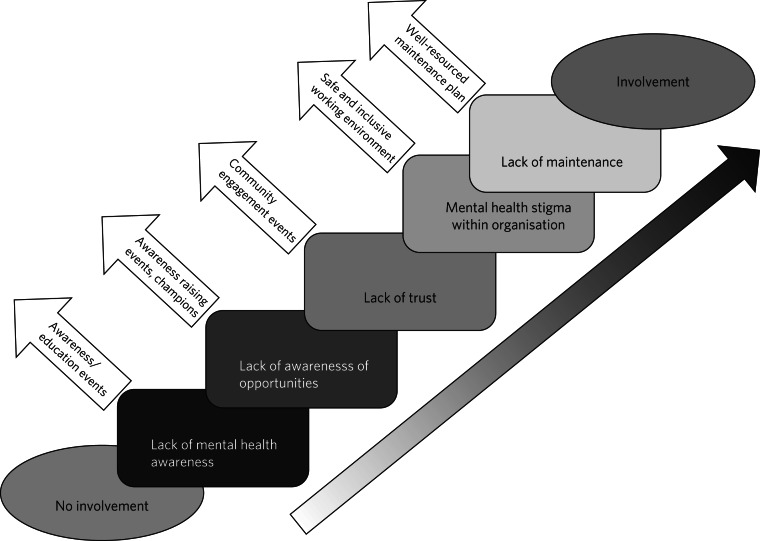
Barriers and facilitators to involvement in mental health professional education.

Delegates in various groups described similar barriers and facilitators to involvement, which lends credibility to these recommendations. See Supplementary File 1 available at https://doi.org/10.1192/bjb.2024.106 for the data categorised as barriers, and Supplementary File 2 for the facilitators and recommendations. Figure 2 shows the barriers and corresponding facilitators to involvement. The recommendations can be summarised in five broad areas. Each will be discussed with excerpts from the roundtable.

#### Organisations should work with underserved communities to educate and raise awareness about mental health

One of the main barriers to involvement was a lack of awareness of the varied perceptions of mental health in different cultures. Before underserved communities can engage with healthcare and academic organisations, it would be important to raise awareness of what mental health is. In many cultures, there are no conceptually or linguistically equivalent terms to describe mental illness:

‘Not seen as an illness, sometimes the words aren't there’ (Group 2).

Awareness raising must be done in collaboration with these communities, with learning taking place on both sides to build bridges of shared understanding rather than imposing the Westernised perspective:

‘I worked with a group of Bangladeshi women to put on a play of what may happen and this was … an engaging way of getting the women to approach the volunteers’ (Group 6).

#### Organisations should seek to build trust with communities where it has been lost

Many of the groups described the way in which healthcare and academic organisations had lost the trust of underserved communities through previous experiences of discrimination, stigma or bias:

‘The most stigma seems to exist in places you wouldn't expect it - mental health trusts - mental health research’ (Group 2).

Organisations need to engage with communities to rebuild trust. Delegates recommended that organisations be intentional about reaching out to communities and the importance of asking those communities how best to do it, rather than making assumptions:

‘In the past we tended to be passive but this doesn't deliver diversity’ (Group 2).

#### Organisations should think more creatively about the promotion of involvement opportunities

How to raise awareness of involvement opportunities in underserved groups was a topic that dominated most groups’ discussions. Creativity is required by organisations to construct an awareness campaign that focuses on a person from the target community, what they would listen to, where they would go and what they would watch:

‘Creative projects, resource community partners, engagement events to recruit, non-institutional venues e.g. Notts Gallery of Justice, community radio, podcast messages, local authorities – public health, local media – BBC EM Today Central News, involving politicians as messengers and engage communities, use bulletins/newsletters/public face’ (Group 1).

It was suggested that organisations could employ someone with marketing experience. Other groups suggested that individuals may immediately discount themselves by thinking they need qualifications:

‘What about people who haven't engaged in school? May be difficult for some but a high level of literacy isn't required’ (Group 2).

Promotional activity should be clear about the requirements of involvement and the roles that are available. Other ideas included promoting through existing relationships (e.g. primary care providers) or seeking out champions from underserved communities. These could partner with organisations, raising awareness in culturally appropriate ways:

‘Word of mouth is more powerful than a poster’ (Group 6).

#### Organisations should actively seek to create a safe and accepting environment for those from underserved communities

One common suggested barrier was the perceived stigma, bias and discrimination within academic and healthcare environments. For people from underserved communities to get involved, these organisations need to change that culture and demonstrate that they are doing so:

‘Change, in culture, attitudes, beliefs, conditioning, respect in medical professionals is needed’ (Group 3).

Delegates focused on four main areas where organisations could seek to improve their inclusivity: training clinicians/academics in public engagement, making their workforce more representative, training in cultural competence that is more about lifelong learning, and involvement of underserved communities in student training and assessment.

‘We don't invite minorities to student's OSCEs!’ (Group 4).

‘Important to pay attention to how racism operates at the micro-level within interactions, so that students are asked to explore how they analyse their own practices that lead to discrimination’ (Group 5).

But it was also appreciated that culture change takes time:

‘It's all about education and it's going to take a long time’ (Group 4).

#### Resources and procedures should be in place to support and maintain involvement effectively once in place

Another barrier to engagement was the way in which engagement projects were often started, but not maintained, often because the funding or project came to an end, making it difficult to sustain engagement over the long term. This perceived lack of consistency or commitment can damage relationships with communities:

‘We lose people from different cultures when funding runs out in community projects, and we don't ever see those people again. I've often wondered what happened to them’ (Follow-up group).

The maintenance required to sustain diverse PPI included financial investment (consistent processes for reimbursement was crucial) and the investment of staff time in the form of a person dedicated to PPI. This person can offer support and work with lived experience educators to ensure their diversity is valued and maximised for the learning of students and well-being of the person involved.

‘It's important to have someone you trust, could be a clinician or a PPI colleague, to encourage you’ (Group 2).

## Discussion

This paper describes the outcomes of a roundtable event where people with mental health conditions, many from underserved communities, discussed the barriers and facilitators to involvement in psychiatry education with clinicians, researchers and charity representatives. The key findings of the roundtable were that awareness of mental health, and trust in academic and healthcare organisations, need to be raised in underserved communities. Promotion of involvement opportunities needs to be creative, and diverse PPI would be both enabled and maintained by the provision of an inclusive environment and sustained support structures.

This paper was limited in that it focused on the East Midlands region of the UK, and the follow-up focus group only represented one local teaching provider. Other areas of the country will have minority communities that are not represented here, and therefore other community-specific barriers and facilitators to involvement that were not considered. We were also unable to audio record the sessions, so some details in the data were lost. However, this event was successful in bringing together people from different social and ethnic backgrounds and disciplines. The hybrid nature of the event, central location and commitment to proper reimbursement removed barriers to attendance. The high proportion of delegates and facilitators with lived experience of mental health conditions or from an underserved community is a rarity in the literature, and has elicited a detailed and practical list of recommendations for organisations who want to widen the diversity of their PPI.

Other literature suggests that time and awareness are barriers to PPI,^9^ but this roundtable further explored the impact of mental health stigma, cultural perceptions of mental health and coming from a minority community, a ‘triple jeopardy’ to involvement in psychiatry education. The cultural perceptions of mental health is globally recognised in the literature as a barrier to accessing health services,^12,20,21^ and this roundtable has underlined the applicability of this dialogue to the PPI conversation. The solutions to overcoming these barriers will be different to those that apply to the general population. For example, simply increasing awareness of involvement opportunities would not increase rates of involvement if the cultural aspects of mental health and building trust are not also considered.

Organisations changing their mindset is crucial to increasing diversity in PPI in psychiatry education. Delegates were aware that every community has different needs, perspectives and habits.^22^ There is no ‘one size fits all’ approach to recruiting a diverse PPI group. Large organisations, which would include any academic or healthcare organisation worldwide, have a love of processes, systems, procedures and efficiency, yet delegates emphasised valuing creativity, the slow building of trust and personal relationships. Any process that seeks to engage varied cultures must be adaptable, personalised and value long-term over short-term gain.^22^

This change in mindset must be accompanied by resources. Research organisations like the National Institute for Health and Care Research in the UK and the Patient-Centred Outcomes Research Institute in the USA are now realising the importance of embedding PPI within the research cycle and budgeting for PPI activity in research proposals.^23,24^ It is important that medical education establishments and their national governing bodies also recognise the importance of sustained resourcing of community outreach activities. Indeed, lack of sustained resources – where relationships are built, but not funded to continue – is more likely to be a long-term barrier, damaging trust that has been built. For the involvement of underserved communities, there needs to be groundwork, which is costly in time for clinicians and financially in reimbursement for time/transport/consumables for members of those communities. Commissioners/medical education directors/team leads need to be aware of this and ensure that funds are appropriately allocated on an ongoing basis.

Organisations need to not only think about how to bring more diverse PPI members into the teaching context, but also how to ensure that the organisation is a safe and supportive environment. An organisation that prizes cultural competence as a lifelong developmental process, as the European Psychiatry Association advocates,^14^ has a workforce that is representative of the local population and has a track record of acting on discrimination and making services accessible, is more likely to be approachable for those interested in involvement. Culture change takes time, and the involvement of patients from a diverse background is a factor that would contribute to that change, but it is clear from this roundtable that an organisation with a passive attitude will never bring about culture change. Organisations need to take the initiative and be active in their pursuit of diverse PPI.

**Fig. 3:**
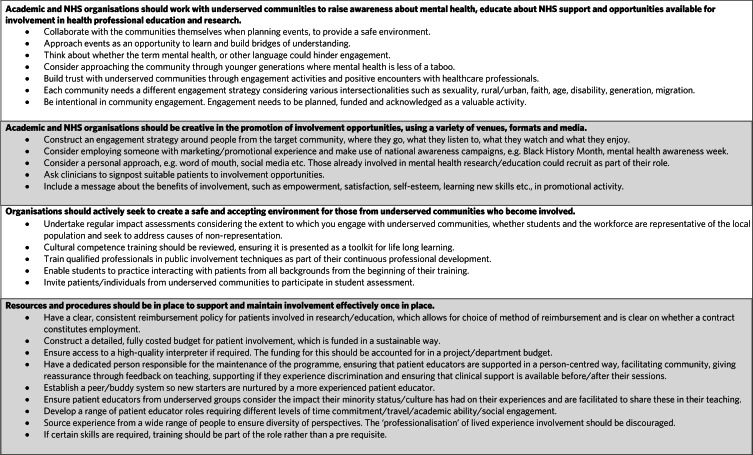
Recommendations for increasing the diversity of patient and public involvement in psychiatry education. NHS, National Health Service.

In conclusion, this roundtable event, attended by people with lived experience of mental health conditions, people from underserved communities, academics, clinicians and charity representatives, has produced recommendations (see Fig. 3) that will be constructive for those involved in mental health professional education, both in the UK and internationally.

## Supporting information

Stanyon et al. supplementary material 1 Stanyon et al. supplementary material

Stanyon et al. supplementary material 2 Stanyon et al. supplementary material

## Data Availability

The data that support the findings of this study are available from the corresponding author, M.S., upon reasonable request.

## About the authors

**Miriam Stanyon** is the Research Project Manager at Derby Psychiatry Teaching Unit, Derbyshire Healthcare NHS Foundation Trust, Derby, UK. **Nagina Khan** is a senior postdoctoral researcher with the Centre for Health Services Studies at the University of Kent, Canterbury, UK. **Analia Buckley** is a lived experience facilitator at Derby Psychiatry Teaching Unit, Derbyshire Healthcare NHS Foundation Trust, Derby, UK. **Naina Patel** is Public Involvement Lead with NIHR Research Support Service Hub, delivered by University of Leicester and Partners, Leicester, UK. **Kirit Mistry** is Chairman of South Asian Health Action, Leicester, UK. **Karl Ryan** is an expert patient at Derby Psychiatry Teaching Unit, Derbyshire Healthcare NHS Foundation Trust, Derby, UK. **Subodh Dave** is Dean of the Royal College of Psychiatrists, a consultant psychiatrist and Deputy Director of Undergraduate Medical Education at Derby Psychiatry Teaching Unit, Derbyshire Healthcare NHS Foundation Trust, Derby, UK.
