# Supplementary material for: Diversity in psychiatry education and patient and public involvement: roundtable analysis
Source: BJPsych Bull. 2026 Feb;50(1):65–71. doi: 10.1192/bjb.2024.106 (PMC13150550; doi:10.1192/bjb.2024.106)
Supplement: Stanyon et al. supplementary material 1 — Stanyon et al. supplementary material [file S2056469424001062sup001.pdf]

Supplementary file 1. Barriers to involvement in psychiatry education

Summary	Excerpt/note from round table	Group
Cultural representations of mental health:<br>Mental health stigma and self-stigma is a barrier so those from underserved communities may avoid situations that focus on it. In some cultures, mental illness is not seen as a medical illness, or language does not exist to communicate about mental illness in the same way. Language used to describe involvement may also be a barrier, e.g. the term Expert Patient, because they do not want to be labelled a patient or they do not think of themselves as an expert.	Stigma	1
	Communication – lack of voice and awareness	1
	Can be shameful, things get hidden away	2
	Would have been great to have a black man here today, but this is a massive taboo for them	2
	Mental health stigma, people in certain cultures don't want to talk about it	2
	Mental health is a taboo subject	3
	Reluctance to accept mental health issue and ask for help	4
	Stigma re mental health	4
	Stigma and self-stigma (ashamed of having a mental health issue/self esteem)	4
	Avoidance of any situation where mental health is the focus e.g. workshops, surgeries etc.	4
	Alternative conceptions of mental illness e.g. possession by evil spirits	2
	Not seen as an illness, sometimes the words aren't there	2
	We need to recognise baseline varies. Used to do regular session in B'ham exploring what is mental health. For a lot of them, don't see any relationship with the organised system so agree need to think of novel ways of engaging with people. We don't have any organised way in medical education of engaging with the community. Should become a requirement of nurse / GP to develop skills of working with community. In terms of barriers, we need to expand our horizons from the UK to other countries, e.g., Spain has a very different community spirit where children run into other houses. This means developing links.	6
	Other barriers might include – cultural variations in world view about mental ill-health…and the risk of being seen as identifying with the aggressor for some	6
	Term Expert Patient doesn't sit comfortably, It excludes people, it comes across arrogant, hierarchical. Who is to say who is an expert patient or not? Psychiatrist, psychologist, nurse, social worker?	Follow-up
Lack of knowledge about involvement opportunities:<br>Communities do not know about involvement opportunities and may have the wrong expectations of what involvement means.	Not knowing where to start. Where do you go to find out what's going on out there?	2
	No awareness of what expert patients are	2
	People may have odd expectations of what's involved in an education setting – may expect they are being expected to teach What participation involves must be very clear	2
	What about people who haven't engaged in school? May be difficult for some but a high level of literacy isn't required. May expect that high level of education is required	2
	Some expert patients aren't comfortable giving written feedback, so the option of giving verbal feedback is available at [teaching organisation]	2

Supplementary file 1. Barriers to involvement in psychiatry education

	Discrepancy between what's offered and what people may perceive – therefore it's a question of identifying potential participants and selling it to them? Need to consider all of the protected characteristics in the Equality Act	2
	According to one of the participants her experience of participating in medical education was by accident it was not promoted widely to diverse ethnic communities so there is a lack of knowledge about the opportunities that exist. So only people that are 'in the know' have the opportunity to take part.	5
	Other barriers might include…lack of awareness of opportunities to get involved, not knowing what involvement means	6
Lack of trust in organisations: Experience of/belief in institutional stigma/discrimination/racism/cultural insensitivity in NHS/academia is a barrier. For many communities trust in organisations e.g. NHS has been lost	Discrimination could be a barrier, past or present - Community engagement could help overcome this	1
	Attitudes	3
	Change, in culture, attitudes, beliefs, conditioning, respect in medical professionals is needed	3
	Bias in research (researchers)	4
	Participants aren't representative so there is research bias So there is a cycle of lack of representation so results aren't representative, so participants aren't representative…	4
	Culturally impose stigma to patients from the community	4
	Culture of the organisation, there is a lack of focus on the how the culture of the organisation impacts negatively.	5
	Issues about racism and discrimination in medical education was only addressed, including sectioning was only addressed with African-Caribbean patient experts but it's something that all students need to be aware of.	5
	The most stigma seems to exist in places you wouldn't expect it - Mental health trusts - Mental health research So people steer clear rather than trying to get involved	2
	Institutional racism Ethnic minorities have heard/experienced racism in NHS/academia and don't want to get involved	4
	Bias in research design Cultural differences need to be taken into account when designing research	4
	Cultural insensitivity Communications or projects may be insulting without realising before they've even tried to recruit anyone	4
	Lack of trust particularly amongst African Caribbean men.	5
	When we think about ethnic minority groups and engaging with services, e.g., fear about social services – all this other stuff will happen but might not have any control, may lose children. This creates a culture in itself that makes it difficult for services to have a positive image of helping. When working in hospital in Yorkshire, personal experience meant that other members of same community with same ethnic	6

Supplementary file 1. Barriers to involvement in psychiatry education

	background, didn't want to reveal confidential information, so took a job outside of area.	
	Lack of education of other services, e.g., police. GP services have changed a lot because of COVID. Patients can hold back if they know that their notes may be used if they are going for a job.	6
	Ethnic minorities have been discriminated on by services, for years, obviously it has affected their views to see services as helpful now. They have been let down, no one's given them information, so they didn't have any proper knowledge about the process of services or positive views.	Follow-up
Lack of engagement by organisations: Organisations don't know how to engage with certain communities and have often not admitted this to themselves. Organisations need to realise they don't know how to engage and be willing to give permission to engage with those who do know.	Educators don't know how to involve minority groups in education	4
	Organisations need to be honest and say we don't know how	5
	"We want to go out in the community, to people with disabilities, religious groups, atheists, temples, mosques, etc ""And have a group that consists of a both professionals and EP Champions on different underrepresented groups/organisations/or communities." "We would need the Trust (NHS organisation) to back this," (EP) "We would need money for transportation, facilities and IT resources for making connections, and training."	Follow-up
Finance:<br>Many people from under-served communities are hindered by the resources required to become involved	Financial Involvement can be expensive e.g. travel, or it could affect benefit entitlement - Expenses should be paid e.g. travel, time sustenance	1
	Financial barrier many people from under-represented communities do not have the finances for involvement, or worry it may affect their benefits.	3
Language:<br>Language is a barrier to involvement, some languages don't even have words for mental health conditions, how mental health is defined is from a Western perspective. Recruitment material is often in English and may contain academic jargon.	Language, signage, interpreters People from under-represented communities may have language difficulties on top of this. Not understanding signage in NHS or academic locations or not having access to interpreters or resource materials (leaflets/communications) in their own language	1
	Communication is a barrier in even getting a diagnosis	3
	Break language barriers Access to interpreters, information in other language formats	3
	Research language isn't accessible So those from ethnic minorities don't understand how they can participate	4
	Language barriers	4
	Language barriers and the quality of the translation	4
	Not seen as an illness, sometimes the words aren't there	2
	Some languages don't have specific words for mental health diagnoses e.g. anxiety and depression so don't even realise it's a health problem	4
	Is the DSM a culturally neutral document? Mental health problems are defined in a Western way. We are	4

Supplementary file 1. Barriers to involvement in psychiatry education

	already coming at the issue from a western viewpoint.	
Maintenance of PPI:<br>It is difficult to maintain patient who get involved because they may find other more attractive opportunities, funding for their post runs out or they are not adequately supported.	Some people move on from involvement because they get worn down, embittered	2
	They move on to other volunteering, many opportunities that are more attractive, less challenging	2
	"This would be better; we lose people from different cultures when funding runs out in community projects, and we don't ever see those people from different communities again. Ive often wondered what happened to them and their health because people need support.	Follow-up
Other barriers: transport, lack of confidence, isolated people aren't involved in community groups, involvement often involves availability in daytime, a person's mental health, in some cultures PPI is totally counter-cultural. Patients don't want to be seen as experts	Access routes into research and clinical education - Venue Transport can be difficult or accessibility for those who can't drive/have access to a car or public transport	1
	Confidence It takes confidence to go somewhere new, especially if you're there because of a health issue with stigma attached	1
	Realise that people are leading isolated lives even in their own communities, for many the isolation experienced in lockdown was a normal way of living even prior to the pandemic. By asking and involving community organisations only, we are likely to miss people who are not involved in these organisations and living their lives in isolation.	5
	People need daytime availability to take part in medical education.	5
	Health Current health status can be a barrier. Fluctuations in health prevents involvement, not wanting to let people down.	1
	What we observed in Manchester in BAME – very few representation in clinical trials and PPI despite trying hard to get their involvement. Did a feasibility study in South East Asia – very new, wasn't heard of. A big shock to the system that doctors may want to hear patient views. Traditionally there is the hierarchy between doctors and patients. Takes a long time to change their understanding and what PPI and research means.	6
	PPI was opposed by Indian mental health professionals themselves thinking they are not qualified to be involved.	6
	"Most people who tend to take part are articulate but mental health is different. When people don't feel okay, they stop talking and don't talk'. "I didn't talk when I wasn't feeling good, my mental health condition makes me stop doing that. Also, when I did want to speak, I wanted to speak to a woman and not necessarily a male professional".	Follow-up
