# Supplementary material for: Diversity in psychiatry education and patient and public involvement: roundtable analysis
Source: BJPsych Bull. 2026 Feb;50(1):65–71. doi: 10.1192/bjb.2024.106 (PMC13150550; doi:10.1192/bjb.2024.106)
Supplement: Stanyon et al. supplementary material 2 — Stanyon et al. supplementary material [file S2056469424001062sup002.pdf]

Supplementary file 2. Facilitators and recommendations according to barriers

Barrier to overcome	Group	Excerpt/note from round table	Summary of facilitator	Recommendation
Cultural representations of mental health:<br>Mental health stigma and self-stigma is a barrier so those from underserved communities may avoid situations that focus on it. In some cultures, mental illness is not seen as a medical illness, or language does not exist to communicate about mental illness in the same way. Language used to describe involvement may also be a barrier, e.g. the term Expert Patient, because they do not want to be labelled a patient or they do not think of themselves as an expert.	1	Awareness of communities about mental health	Awareness about mental health, mental health education and research needs to be raised before people can be involved	Academic and NHS institutions should work with underserved communities to raise awareness about mental health, to educate about the help on offer by the NHS systems and the opportunities that are available for involvement in mental health education and research.<br><br>The way in which this is done must be in collaboration with the communities themselves so that the environment is seen to be safe and so that there is real engagement from the community rather than the nature of this awareness raising being purely dictated by institutions.<br><br>Institutions need to approach these engagement events with the intention of learning from communities about how they perceive mental health in order to bridge gaps.
	2	People could benefit from some training or awareness raising before becoming an expert patient - What would this involve? Assurance that this is safe. There are no rights and wrongs		
	5	Facilitate time and space for African-Caribbean communities to talk amongst themselves about their lives and their communities. Work needs to be done within communities to create awareness of mental health first		
	6	Is a lot of meeting people in terms of where their safe space is and demystify research and explaining what outcomes can look like. Regards MH there is a lot of taboo and stigma. My personal view is that there needs to be a lot of community work to be done. Need to be innovative. Has worked with a group of Bangladeshi women to put on a play and a scenario of what may happen and this was a useful way in to talk about the characters. That was an engaging way of getting the women to approach the volunteers. Need to go to them and break out of comfort zone of university setting.		
	6	We need to recognise baseline varies. Used to do regular session in B'ham exploring what is mental health. For a lot of them, don't see any relationship with the organised system so agree need to think of novel ways of engaging with people. We don't have any organised way in medical education of engaging with the community.		
	6	What training and support can be offered in the pre-step. Also benefits shared for the importance of this work to some of the underserved populations.		
	6	In the past women haven't taken part, we need inclusion at every level. If we work holistically, we need to work with them where they are at. We can bridge these gaps. We can offer continuity.		
	Follow-up	"We need to get to know each other to really support people in the community." (EP)		
	5	Might we get a better response if we call it wellbeing rather than mental health, we could get a better response.	Call it well-being rather than mental health	Institutions must consider whether the term mental health, patient (or other language used) could hinder engagement, and consider how best to frame the conversation to enable engagement.
	5	A contradictory view to the above was that amongst younger people, mental health as a word and concept is being normalised. There is so much discussion amongst young people about self-harm	Mental health may be less taboo for younger people	When trying to engage a community with mental health issues it may be easier to approach through the younger

Supplementary file 2. Facilitators and recommendations according to barriers

		and suicide ideation however, stigma and taboo about mental maybe greater amongst older people.		generations where mental health is less of a taboo.
	5	If people knew and were educated about mental health illness maybe more might come forward. Young people are more accepting of mental illness due to social media as there are forums on bi-polar.		
	5	Organisations should be looking at how younger people can be involved and inspire organisations.		
Lack of trust in organisations: Experience of/belief in institutional stigma/discrimination/racism /cultural insensitivity in NHS/academia is a barrier. For many communities trust in organisations e.g. NHS has been lost<br>Lack of engagement by organisations:<br>Organisations don't know how to engage with certain communities and have often not admitted this to themselves. Organisations need to realise they don't know how to engage and be willing to give permission to engage with those who do know.	1	Support - Trust needs to be developed between individuals and organisations	Building trust between organisations and communities is important. See how other organisations have built trust.	Institutions should seek to build trust with underserved communities through engagement activities and positive encounters with healthcare professionals.
	3	Gain trust back – identity Many under-represented groups have had poor experiences of NHS and will worry about the same happening again. May not be willing to be vulnerable		
	3	Gain trust Organisations need to do what they say they're going to do		
	3	Working on empathy and trust, doing more in earning the trust of under-represented groups		
	6	Building trust is really important		
	Follow -up	Example: "When I was growing up as a brown person, as a young girl a police commissioner came to the Asian girls' group that I attended and well that had a huge impact on me" (EP)		
	1	More consultation with the underserved groups	Ask those communities and don't make assumptions	Work to engage underserved communities must be done with those communities, asking them how best to do it.
	2	No representation – open up, speak up, gain knowledge, break the chain		
	4	Is engagement with minority groups developed with minority groups? It is people from the minority groups themselves who know how to recruit from minority groups. They need to be involved from the start		
	6	Diversity in mental health – need to think of all groups beyond BAME. We can be inclusive to ask communities how we can make it accessible to them rather than making assumptions.		
	1	With all of this it's not one size fits all Reasons will be different for different under-represented groups	Engagement strategies are not one-size fits all<br>not just racial communities but rural/urban, faith communities, age groups	Each community needs a different engagement strategy. Differences are not simply racial but other intersectionalities should be considered such as sexuality, rural/urban, faith, age, disability.
	3	Race specific and under-represented groups Different strategies required for different communities		
	3	Provide training to specific communities Not one size fits all		
	5	Key message: do adopt the assumption that mental illness is stigmatised within all and across all ethnic groups because there are differences.		
	6	Identity that an individual has; PPI may work well in one area of the UK but might not in another and really understanding the fears and		

Supplementary file 2. Facilitators and recommendations according to barriers

		lack of awareness of a particular issue. We need to do some pre-PPI work to try to understand some of the challenges.		
	5	Develop different messages for younger and older people.		
	5	Be aware of different communities such as rural/urban and faith communities. It's not just about ethnic background but location, religion, socio-economic status		
	1	Institution - Community engagement the institution itself could engage with communities	engagement must be intentional	Institutions should be intentional in their community engagement. Engagement should be planned, funded and acknowledged as a valuable activity that will improve services and the nation's health.
	1	Discrimination could be a barrier, past or present - Community engagement could help overcome this		
	2	To get more diversity need to plan more needs to be intentional and it takes work		
	2	In the past we tended to be passive but this doesn't deliver diversity		
	3	All parties need to be proactive		
	3	Diversity – Going to communities Initiative must be taken from the organisation's side (NHS/ Universities)		
	3	Better understanding On both sides. Organisations need to better understand minority groups and barriers. Minority groups need to better understand mental health		
Lack of knowledge about involvement opportunities: Communities do not know about involvement opportunities and may have the wrong expectations of what involvement means.	1	Information/ opportunities - Different format E.g. does it all have to be written, what about recruiting through radio/word of mouth	To recruit, organisations need to: Promote widely, use different venues/formats/social media and technology to advertise e.g. Carnivals, takeaways, radio, TV, podcasts, Black history month, Present at conferences, Bollywood event, A national campaign similar to the one run by NHS England where athletes shared about mental health. Institutions should consider employing someone with promotional/marketing experience. Reach people where they're at and where they feel comfortable, where do they go? What do they listen to? What do they watch? What do they enjoy?	Academic and NHS institutions should think more creatively about the promotion of involvement opportunities using a variety of venues, formats and media.<br><br>Institutions should think about the people they are trying to reach and construct an engagement strategy around where people from that community go, what they listen to, what they watch and what they enjoy.<br><br>Institutions should consider employing someone with marketing/promotional experience to be intentional about recruitment.<br><br>Institutions could make use of national awareness campaigns e.g. Black History
	1	Creative projects		
	1	Resource community partners		
	1	Engagement events to recruit		
	1	Non Institutional venues e.g. Notts gallery of justice		
	1	Community radio		
	1	Podcast messages		
	1	Local authorities – public health		
	1	Local media BBC EM Today Central news		
	1	Involving politicians as messengers and engage communities		
	1	Use bulletins/newsletters/public face		
	2	Promotional experience Employ someone with promotional experience on the team		
	2	Access and knowledge = marketing campaign. Cultures, religion, taboo subjects		
	2	Better advertising and promotion		
	3	Spread the information Don't just have one strategy, think wide		
	3	Can we duplicate what has worked in other areas and bring to under-represented areas?		

	5	Consider using different methods of communication such as Carnivals, takeaways to take the message out to different people such as young people.		Month, PRIDE week, mental health awareness week.<br><br>Institutions can consider other campaigns that have been successful and seek to apply these methods to their own context.
	5	Promote widely opportunities to participate in medical education otherwise how are students going to learn how to support, for example, African-Caribbean communities.		
	6	Have to think outside the box. When I am recruiting for patient group, get the people who have the spare time to come to an evening meeting. With technology we can now use social media etc. to reach people where they are.		
	5	Also, hold events such as black history month.		
	6	Working at CCG as a practice manager conference and did a presentation about research and the importance of PPI and how important and valuable it is, got interest. Used lay language. Got to reach people at where they are at.		
	6	Previously organised a Bollywood event with a grant from RCPsych – was a good way of engaging with the community. Some of the expert patients wanted to be involved in the future, it was difficult for them to find a way to network.		
	6	A shame we can't have a national campaign run by NHS England, like some of the athletes that have shared their story of mental health. This would help reduce the stigma.		
	5	Healthwatch (don't know which region) do it well, lots of interactive work and supportive feedback. Based on the feedback they received they changed the way in which their organisation provides feedback.		
	Follow -up	People then tend to be different and behave or respond differently, they tell you things that they wouldn't in a clinical setting, things that are important and precious to them and their health. They feel they aren't judged.		
	Follow -up	"What we should be doing is going to the groups that aren't coming to us" What to do and try: Social Media messages to inform and increase knowledge and engage people from specific communities. Local radio, Community speakers, who are visible. Engaging the arts community because they are more informal using informal stings and informal groups for information dissemination. If we do nothing things will stay the same for a lot of people who need help and support. We need to be outside the services, outside on the floor. Walking groups Community theatre groups		
	2	Existing expert patients have an important peer role to help in recruiting new expert patients	Those already involved can get others involved but they will	Institutions should also consider a narrow approach as well as a broad

	Follow -up	“We are used as Guinea pigs, we want to change things, learn, EPs should tell their story of about how they got there and who these people were”	need the permission and support from their organisation.	approach. Those already involved in mental health research/education should consider recruiting others as part of their role. In partnership with healthcare professionals, existing expert patients can be a bridge to under-represented communities. Institutions need to support these champions and appropriately resource them with finance and training. This is a way of changing current healthcare for others.
	Follow -up	“I'm hearing that EP participation is not good enough, you want to be part of change, genuine change to help others, and you want to be part of implementation and the success of it.” “EPs want to make a difference to the process for others, influence medical education, so others don't get the response they did to change something for others.”		
	Follow -up	“We should as EPs, be champions and go out in the community.”		
	Follow -up	“We want to go out in the community, to people with disabilities, religious groups, atheists, temples, mosques, etc ” (EP)		
	Follow -up	“And have a group that consists of a both professionals and EP Champions on different underrepresented groups/organisations/or communities.		
	Follow -up	“We want to go out in the community, to people with disabilities, religious groups, atheists, temples, mosques, etc ”“And have a group that consists of a both professionals and EP Champions on different underrepresented groups/organisations/or communities.” “We would need the Trust (NHS organisation) to back this,” (EP) “We would need money for transportation, facilities and IT resources for making connections, and training.” “Training around appropriateness in communicating with different cultures and knowing what’s appropriate or not”. (EP) “We don't want to offend anyone” (EP)		
	Follow -up	“We should have different sessions for every community in education” (EP) “Might not be able to do that realistically, in a formal educational setting?” (Educator) “Well, what's the point then, what's the point of all this” (EP) “We have 60 EPs. That can go out to the community and set up teaching sessions in the community groups/ we could enable enough teaching sessions.” (EP Programme Facilitator) “It needs to happen” (EP)		
	Follow -up	“We need resources to do this, everybody needs a session on working in the community and its needs if they aren't being represented.” (EP) We need: “Bigger teams, More teaching sessions, Group of experts going into the communities, working with community experts, and professionals, to break down, barriers, EP's to be trained as champions, Even people who aren't so articulate, People who don't engage, Openness in informal situations		

Supplementary file 2. Facilitators and recommendations according to barriers

	Follow -up	"We should also look at pathways for different people who came onto the EP programme. Like pathway stories, how did they come to be Eps and why them and not others from other groups, that we don't tend to get."	Think about which methods of recruitment have worked for whom and evaluate recruitment techniques to target under-represented populations.	Utilise recruitment techniques which will target under-represented groups.
	Follow -up	"We need to look at the data to see which groups we don't really get, because some of the groups that were highlighted in the previous hybrid sessions talked about black men and women. We think here we may have different groups that aren't represented."		
	1	Information/ opportunities - Cultural adaptation Ask someone from that community to adapt materials/communication etc to culturally acceptable/accessible format	Get members of those communities (research ambassadors) to reach others in that community. But these must be chosen with care to be those who will truly be ambassadors and not simply seeking a platform for complaints about their own treatment.	Institutions seek out an ambassador within the underserved community who can co-produce events/materials and approach other members of that community from a place of knowledge about that community and a wish for bridges to be built between that community and institutions.
	5	Consider the use of research ambassadors and reach out to communities.		
	6	Has worked with a group of Bangladeshi women to put on a play and a scenario of what may happen and this was a useful way in to talk about the characters. **That was an engaging way of getting the women to approach the volunteers**. Need to go to them and break out of comfort zone of university setting.		
	2	Word of mouth is very powerful		
	6	Positive case studies, people getting treatment that helped, then people would find out that good things can also happen in services. Word of mouth is more powerful than a poster and help build trust to use services.		
	Follow -up	Role models from communities Well they start off well and then its more about them than the issue, which doesn't actually help others anymore. Example was given, whereby an individual from an ethnic minority group opens about their struggles, at the beginning its great and then it just seemed like they are just 'showing off.' No 'Subtlety' because people lose respect and then no one is interested. Do we effectively break down any barriers?		
	1	Institution could be a barrier - Clinical support (CPN) could help to ease a transition, should be told about involvement	Contact through primary care services	Opportunities for involvement could be promoted through primary care services as there is often more of a trusting and longer-term relationship.<br><br>Clinicians can signpost suitable patients to involvement opportunities in psychiatry education.
	6	I also think primary care could be involved in engaging patients in PPI as they usually have involvement longitudinally and patients trust primary care than secondary care services		
	6	We need to capitalise on positives e.g., GPs often have trust with the community. PPI is essentially a leadership role. To help others realise they could have better care as people do fear readmission.		
	6	Under-represented areas do have access to healthcare services, a health visitor, dr, CPN will all have contact. Whatever the health		

Supplementary file 2. Facilitators and recommendations according to barriers

		professional says, is like gold. If we use the right approach, and if they are made aware, they will be included.		
	2	Clinicians are well places to signpost people to become expert patients		
	2	Volunteering can be good for self-esteem	Send the right message about involvement by talking about positive cases, mutual benefits, that it's empowering and good for self esteem	Promotional activity should include a message about the benefits of involvement such as empowerment, satisfaction, self-esteem, learning new skills etc.
	2	Mutual benefits, helping students is often emphasised but improving wellbeing of expert patients is less talked about.		
	6	Positive case studies, people getting treatment that helped, then people would find out that good things can also happen in services. Word of mouth is more powerful than a poster and help build trust to use services.		
	6	Messaging around the role of PPI should show that it's empowering, collaborative and more satisfying for both patients and providers		
Lack of trust in organisations: Experience of/belief in institutional stigma/discrimination/racism /cultural insensitivity in NHS/academia is a barrier. For many communities trust in organisations e.g. NHS has been lost	3	These are barriers in medical professionals. Medical professionals need to change these things before barriers can be taken away. The following are ways in which medical professionals could change to remove these barriers	Clinical professionals need training in public involvement/racism awareness	Institutions have a responsibility to undertake impact assessments which also take into account the extent to which they engage with underserved communities<br><br>As part of cultural competence/communication skills training, mental health professional students should be encouraged to analyse racism/discrimination taking place at the micro-level in interactions<br><br>Review cultural competence training to ensure it is not presented as a qualification to be achieved but a toolkit to use when interacting with other cultures, to help uncover cultural biases and learn to see mental health from the point of view of the patient and to help with life-long learning.
	3	Look at the issues with 'non-white' eyes, how would you deal with it		
	3	Changing the narrative to accommodate us		
	4	Train 'professionals' in involvement Train public in involvement Train together Everyone needs to learn to do things differently		
	5	Need for organisations to undertake equality impact assessments, their training and delivery, analyse the way in which students perceive their patients.		
	5	Do something specific in addition to race awareness training, the latter is generic but fails to focus on specifics such as the way in which a student interacts with patients is not taken into account.		
	5	For professionals to realise their own prejudices.		
	5	Important to pay attention to how racism operates at the micro-level within interactions, so that students are asked to explore how they analyse their own practices that lead to discrimination. This opportunity to focus on micro-level of discrimination and interactions is missed.		
	6	Should become a requirement of nurse / GP to develop skills of working with community.		Already qualified professionals should be trained in public involvement techniques as part of their CPD.
	3	Education: Theory and lived experience (early practice experience) leads to excellent medical professionals early exposure to lived experience will lead to better medical professionals	students need early exposure to patients of all backgrounds but change takes time	Students should be able to practice interacting with patients from all backgrounds as early as possible.

Supplementary file 2. Facilitators and recommendations according to barriers

	3	Investment in stages of education at the basic level, while still students, before climbing the ladder e.g. training in bedside manner and language used by medical practitioners		Patients involved in education should be from diverse backgrounds.
	4	It's all about education and it's going to take a long time Culture takes a long time to change		
	4	We don't invite minorities to student's OSCEs So medical students aren't ever assessed on their ability to communicate/relate to/treat people from minority groups		Patients/individuals from underserved communities should be invited to participate in healthcare professional student assessment
	1	Representation - More diversity of students in mental health education If the students were more diverse it may encourage more diverse people with lived experience	a greater diversity of workforce would help make others more comfortable	Institutions should work to ensure that their students and workforce is representative of the local population and seek to address any causes of non-representation
	1	Institution - Diversity of staff could help with involvement. If there are people already working there like me		
Finance:<br>Many people from under-served communities are hindered by the resources required to become involved	1	Financial Involvement can be expensive e.g. travel, or it could affect benefit entitlement - Expenses should be paid e.g. travel, time sustenance	expenses should be paid in a way that doesn't affect benefits	Institutions should have a clear, consistent reimbursement policy for patients involved in research/education. The policy should allow for patient choice as to the method of reimbursement so that benefits are not affected.<br><br>Any reimbursement policy must be clear on the nature of any contract whether it constitutes employment or not.<br><br>Any project/department must plan a detailed, fully costed budget for patient involvement showing that lived experience involvement is planned with as much detail as the work of academically/clinically trained team members.<br><br>The 'professionalisation' of lived experience involvement should be discouraged. Public involvement should seek to source experience from a wide range of people to ensure diversity of perspectives. If certain skills are required,
	2	Remuneration is important, can affect benefits		
	6	Reimbursing expenses when someone participates in engagement.		
	2	The institutes responsibility is to have and develop policy on what gets paid for and what doesn't	institutions must have a clear reimbursement policy	
	1	Financial - Consistency of payment Payment for different things differs greatly, can be vouchers or cash or bank transfer. Things change when project stop and start. A consistent policy recognised by job centres etc. would help.	payments and method of payments should be consistent	
	2	Clarity on status as to whether a person is employed or not is important from the outset. There are examples (uncommon) of people doing involvement in multiple places to make a full-time job.	needs to be clarity on whether a person is 'employed'	
	6	Funding opportunities is a critical point. NIHR have some funds to build in PPI - increasingly funders recognise this. Often its not fully costed. Funders need to recognise the value and the cost. It is costly and it should be valued as the same value as researchers involvement, in terms of timeline and budget.	projects must budget for financial compensation for volunteers	
	2	If you professionalise involvement you may exclude the most vulnerable people (not typical), and these are the most relevant (from under-served backgrounds)	important the role doesn't become professionalised	
	2	Being an expert patient shouldn't be a job as such, just expenses		
	2	Risk of losing important people if you professionalise the expert patient role		

Supplementary file 2. Facilitators and recommendations according to barriers

				training should be part of the role rather than a pre-requisite.
Maintenance of PPI:<br>It is difficult to maintain patient who get involved because they may find other more attractive opportunities, funding for their post runs out or they are not adequately supported.	1	Support - Buddy of similar lived experience A buddy who understands the condition	Enable relationships with others in similar background	Once involvement has begun organisations should establish plans for the maintenance of the patients involved by: having a dedicated person in the organisation who is responsible for the maintenance of the patient involvement programme, ensuring that patient educators are supported in a person-centred way, facilitating networking between those with lived experience, giving reassurance through feedback on their teaching, ensuing that they don't experience discrimination/are supported if they do, ensuring that clinical support is available before/after their episodes of involvement in case of distress.
	1	Support - Network with others of a similar condition or of a similar background would help involvement		
	1	Support would help people to be involved - Family If family could be informed and be on board that would enable better involvement in some communities	involving their family (for Indian families)	
	1	Support - Reassurance	reassurance	
	3	Reassurance Maintain contact, keep talking		
	5	It's important to provide feedback, follow up and being valued.		
	1	Discrimination could be a barrier, past or present - Community engagement could help overcome this	making it a safe place	
	2	How can we attract more people? - Safe place, need to look after people and support them properly		
	2	Being able to visit the teaching centre is very useful To get to know the place so it doesn't feel so scary		
	2	How can expert patients be encouraged? They need to have had a good experience in the first place.		
	1	Institution - Person-centred care involvement too needs to be person-centred, just like healthcare	be person-centred	
	1	Support - Advocate	having a dedicated person to support	
	1	More organisation, supported with engagement and mentoring support		
	2	Having someone there to support expert patients is important before and after sessions		
	2	It's important to have someone you trust, could be a clinician or a PPI colleague, to encourage you		
	1	Buddies may help to mitigate this and give confidence	peer support/buddy	A patient involvement programme should consider establishing a peer/buddy system so that new starters are nurtured by a more experienced patient educator.
	1	Role models		
	2	'Have you ever thought of becoming an expert patient? I will come with you' Someone who's done it can help bring someone along		
	5	Consider peer support approaches as it is time consuming without support and a recognition of the work that is involved.		
	4	Have a comprehensive syllabus to support involvement	When people get involved, need to make sure their involvement isn't tokenistic and their experience is shared	When patient educators are recruited from minority groups/underserved groups organizations must ensure that patient educators consider the impact their minority status or culture has had
	5	Generic case studies which do not involve a focus on the histories of discrimination and racism will ill-equip expert patients to discuss issues with students. Therefore, it is important for expert patients to be involved in the curriculum and case studies. Medical education		

Supplementary file 2. Facilitators and recommendations according to barriers

		programmes need to allow those from minority communities to speak about differences. Maybe their position as someone from a minority community isn't actually spoken of when they participate?		on their experiences and are facilitated to share these in their teaching. This will enable students to learn from these experiences and avoid tokenistic tick-box recruitment.
	1	Health - Involvement opportunities More opportunities where health fluctuations wouldn't be a problem	Different roles available so instability of health or availability in daytime wouldn't be a problem	Institutions should consider a range of patient educator roles requiring differing levels of time commitment/travel/academic ability/social engagement so that patient educators have options for involvement that are more able to suit their lifestyle.
	Follow -up	"This would be better; we lose people from different cultures when funding runs out in community projects, and we don't ever see those people from different communities again. I've often wondered what happened to them and their health because people need support.	Funding for involvement from minority groups needs to be from a consistent and maintainable source	Institutions should endeavour to fund their recruitment and maintenance of involvement from minority groups in a sustainable way
Language: Language is a barrier to involvement, some languages don't even have words for mental health conditions, how mental health is defined is from a Western perspective. Recruitment material is often in English and may contain academic jargon.	1	Language, signage, interpreters People from under-represented communities may have language difficulties on top of this. Not understanding signage in NHS or academic locations or not having access to interpreters or resource materials (leaflets/communications) in their own language	have a good quality interpreter available	When wanting to involve people from underserved communities there should be access to a high-quality interpreter if required. The funding for this should be accounted for in a project budget.
	4	Language barriers and the quality of the translation		
